# On the Evolution of Handedness: Evidence for Feeding Biases

**DOI:** 10.1371/journal.pone.0078967

**Published:** 2013-11-13

**Authors:** Jason W. Flindall, Claudia L. R. Gonzalez

**Affiliations:** The Brain in Action Laboratory, Department of Kinesiology and Physical Education, University of Lethbridge, Lethbridge, Alberta, Canada; University of Kansas, United States of America

## Abstract

Many theories have been put forward to explain the origins of right-handedness in humans. Here we present evidence that this preference may stem in part from a right hand advantage in grasping for feeding. Thirteen participants were asked to reach-to-grasp food items of 3 different sizes: SMALL (Cheerios®), MEDIUM (Froot Loops®), and LARGE (Oatmeal Squares®). Participants used both their right- and left-hands in separate blocks (50 trials each, starting order counterbalanced) to grasp the items. After each grasp, participants either a) ate the food item, or b) placed it inside a bib worn beneath his/her chin (25 trials each, blocked design, counterbalanced). The conditions were designed such that the outward and inward movement trajectories were similar, differing only in the final step of placing it in the mouth or bib. Participants wore Plato liquid crystal goggles that blocked vision between trials. All trials were conducted in closed-loop with 5000 ms of vision. Hand kinematics were recorded by an Optotrak Certus, which tracked the position of three infrared diodes attached separately to the index finger, thumb, and wrist. We found a task (EAT/PLACE) by hand (LEFT/RIGHT) interaction on maximum grip aperture (MGA; the maximum distance between the index finger and thumb achieved during grasp pre-shaping). MGAs were smaller during right-handed movements, but only when grasping with intent to eat. Follow-up tests show that the RIGHT-HAND/EAT MGA was significantly smaller than all other hand/task conditions. Because smaller grip apertures are typically associated with greater precision, our results demonstrate a right-hand advantage for the grasp-to-eat movement. From an evolutionary perspective, early humans may have preferred the hand that could grasp food with more precision, thereby maximizing the likelihood of retrieval, consumption, and consequently, survival.

## Introduction

Previous research has indicated that the defining characteristic of handedness, that is, a lateralized manual hand preference, does not develop until 21 months of age in humans [1]–[3]. Many manipulative tasks do not show lateralization until much later in development [4]–[7]. With few exceptions, children younger than 4 years of age do not demonstrate a hand preference for manipulative reach-to-grasp actions on non-food objects; instead, they use whichever hand is ipsilateral to the target object [8], [9]. Recently, however, it has been shown that when the target is a food-object, children as young as one will demonstrate a robust right-hand preference for the reach-to-grasp action [10]. In the Sacrey et al. study, 3- to 5-year-old children were presented with food items (Froot Loops®) and non-food items of comparable size and colour (LEGO® construction blocks) and their hand preference for reaching-to-grasp the items was recorded. A right-hand preference for grasping the blocks was found in the 4- and 5-year old cohorts, but *not* in the 3-year old group; this finding was consistent with previous research [8], [9]. When the target was a food-object, however, 3-year-olds showed a greater than 80% right hand preference for grasping. In fact, when younger groups were tested, this preference was observed in children as young as one year of age [10]. This finding suggests that a right hand preference for reach-to-grasp for food (henceforth referred to as grasp-to-eat) develops earlier, and perhaps is altogether *separate* from hand preference for reach-to-grasp for objects to manipulate (i.e., grasp-to-place). This suggestion is further supported by studies which show that infants are able to produce accurate hand-to-mouth movements earlier than accurate reach-to-grasp movements [11]. If hand preference is susceptible to the end goal of an action, it is reasonable to speculate that kinematics may also vary according to the actor’s intent.

Several studies describing the kinematics of prehension have shown that the end goal of an action significantly influences the kinematics of the reach and grasp [12]–[16]. In movements with a similar initial lifting phase, but different consecutive movements (i.e. with differing intent; for example, grasp-to-*place* versus grasp-to-*throw*), peak velocity, peak deceleration, and peak grip aperture of the approach phase of the grasp movement have been shown to vary according to the purpose of the grasp [12]. While food has been used as a target in kinematic analyses [17]–[21] and imaging studies [22], few have investigated whether action intention influences movement kinematics when grasping a food item. In the only such study (of which we are aware), participants were asked to reach and grasp a sugar cube in order to put it in their own mouths (presumably to eat), in the mouth of another person (i.e. a conspecific), or in a fake mouth placed over their own mouths. The results showed greater automaticity for movements directed to the self than either the conspecific or the fake mouth [23]. These studies indicate that the final objective or purpose for which a reach-to-grasp action is executed significantly influences the kinematics of the movement. They also demonstrate the sensitivity of kinematic parameters when detecting differences in seemingly similar actions.

Investigations into the kinematics of left- versus right-handed movements have shown, at most, only minor differences between the hands in reach-to-grasp actions [24]–[27]. For example, [24] used a reach-to-grasp task in which a cylindrical object was grasped and placed into a target slot to compare kinematic data between the left and right hands of participants. Other than a minor difference in insertion time (in which the dominant hand was faster than the non-dominant hand), the researchers found no significant differences in movement kinematics between the hands. This is quite puzzling given that, if the right hand is used preferentially for the grasp-to-place action, and this preference is to be driven by a kinematic advantage, one would expect to find kinematic differences between the hands. The studies cited above, however, have used grasp-to-place tasks in their search for manual asymmetries. Where hand differences are absent in the grasp-to-place movement, perhaps they may be found in the grasp-to-eat movement. This speculation would be supported by research suggesting, first, that prehension originally evolved as a grasp-to-eat action [28], [29] and second, that the right hand preference for grasp-to-eat movements develops years earlier than does the preference for the grasp-to-manipulate (aka grasp-to-place) movement [10].

In the present study, we investigate if the grasp-to-eat action is different from the grasp-to-place action for both right- and left-handed movements. To this end, we measured reach and grasp kinematics of 13 participants who were instructed to reach-for and grasp food items of various sizes to either a) bring the food item to the mouth and eat it (grasp-to-eat), or b) place the food item in a bib located just beneath their chin (grasp-to-place). Both tasks used the same types of food items, required the same types of grasping movement, and differed only in the end-point goal of the movement.

## Methods

### Participants

Thirteen right-handed University students (11 female; average age 20.3 years) participated in the experiment and received course credit for their participation. Handedness was determined through a modified Edinburgh handedness questionnaire [30]. All participants gave informed written consent prior to the onset of the study, in accordance with the principles expressed in the Declaration of Helsinki and with the approval of the University of Lethbridge Human Subjects Research Committee (protocol #2011–022). Participants were able to withdraw from the study at any time without consequence.

### Materials

Three infra-red light emitting diodes (IREDs) were placed on the participant’s hand; two on the distal phalanges of thumb and index finger, slightly proximal with respect to the nails, and one on the wrist at the medial aspect of the styloid process of the radius (proximal and medial with respect to the anatomical snuff box). Two Optotrak Certus camera bars [*Northern Digital, Waterloo, ON, Canada*] recorded IRED position during each trial at 200 Hz for 5 seconds. Vision was restricted between trials using Plato Liquid-crystal glasses [*Translucent Technologies, Toronto, ON, Canada*] worn by the participant throughout the testing session. All experimental equipment was controlled using Superlab 4.5 [*Cedrus Corporation, San Pedro, CA, USA*] and NDI First Principles [*Northern Digital, Waterloo, ON, Canada*].

**Figure 1:**
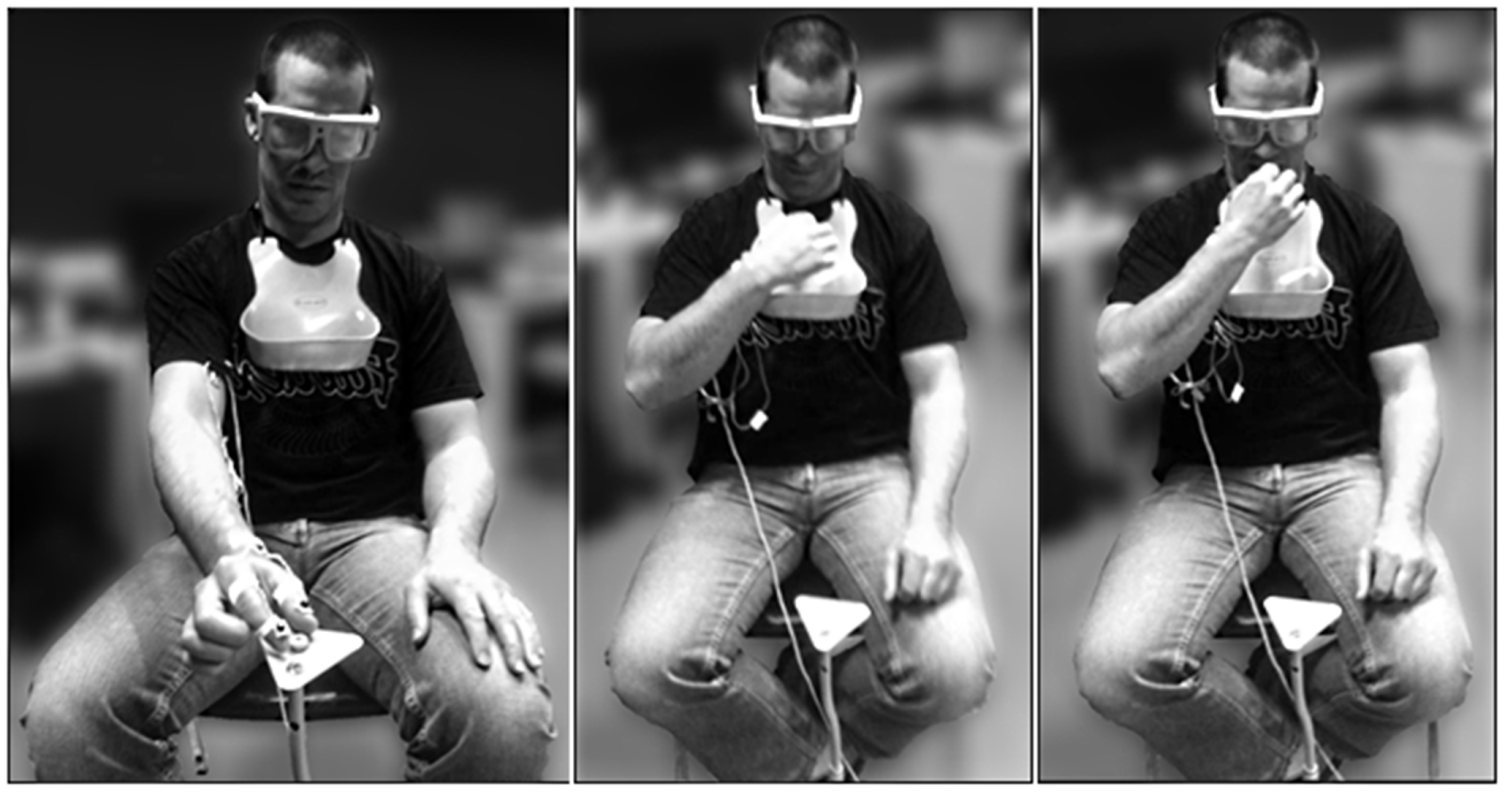
Experimental set-up. *Left:* Participant reaches-to-grasp presented target (item shown: Froot Loop®). *Center:* PLACE task requires participant to grasp the target and place it in the bib hung below their chin. *Right:* EAT task requires participant to eat the target after grasping. Note that participants wear the bib throughout all blocks, regardless of start order or current task, and that all grasps are completed using only the index finger and thumb. The subject of this figure has given written informed consent, as outlined in the PLoS consent form, to publication of these photographs.

Participants were seated before a self-standing height-adjustable triangular pedestal (Fig. 1). The pedestal held individual cereal food items (presented one at a time) of 3 different sizes: SMALL (Cheerios®, mean diameter 11 mm), MEDIUM (Froot Loops®, mean diameter 15 mm), and LARGE (Oatmeal Squares®, mean length 21 mm). These targets were chosen based on their distinct sizes and familiarity. The distance to the pedestal was normalized to each participant’s reach distance (100% of length from shoulder to index finger with elbow at full 180° extension). The height of the pedestal was adjusted for each participant such that the food was at a comfortable reach height (approximately level with the base of the sternum of the seated participant), but also such that the edge of the pedestal did not act as a direct obstacle during the reach-to-grasp movement [31].

### Procedure

Participants sat behind the pedestal, with their hand placed comfortably on their lap (fingertips of thumb and index finger together) between trials. Targets were presented in a pseudo-random order, such that the participant was naïve to the size of the food item until the beginning of the trial, when the goggles transitioned to their transparent state. After 1000 ms of transparency during which the participant had full view of their hand and target, an audible go-signal (‘beep’) was presented, informing the participant that they should begin the reach-to-grasp movement “at a comfortable pace.” After grasping the target between the thumb and index finger in a precision grip, participants would either a) ingest the item completely (EAT condition), or b) place the item in a bib hanging below their chin (PLACE condition) (Fig. 1). Investigators replaced food items between trials, while the liquid crystal goggles were in a closed (opaque) state. EAT and PLACE task conditions were presented in blocks of 25 trials (8 SMALL, 8 MEDIUM, 9 LARGE, randomized), with start order counterbalanced between participants. Participants were informed of task requirements at the beginning of each block. After both blocks were completed, IREDs were transferred to the participant’s other hand, and the process was repeated. Hand start order was counterbalanced between participants.

### Analyses

Kinematic comparisons were made between reach-to-grasp phases of each movement. The time of grasp contact was defined as the point at which: i) the subject’s outward speed dropped below.02 m/s, and ii) their corrected grip aperture plateaued at the approximate diameter of the target. Movement time (MT) was calculated as the difference between time of grasp contact and reaction time (defined as the time following the go signal at which a participant achieved a resultant equal to 5% of their peak velocity) and represents the span during which the participant reached outward toward the target. Peak velocity (PV) was defined as the maximum resultant velocity the participant achieved during their reach towards the target. Deceleration phase duration (DP) was calculated as the time during which the participant was decelerating while still reaching outwards toward the target (time of grasp contact minus time of PV); it is reported as a percentage of total movement time. Maximum grip aperture (MGA) was measured as the peak resultant distance achieved between the thumb and index finger prior to the time of grasp contact. Variability of MGA (vMGA) is the standard deviation of the MGAs of each Hand/Task/Size grouping.

Between Hand comparisons required GA calculations to be corrected for IRED placement, as grip aperture calculations were based on distance between the IREDs, rather than actual distance between subject fingertips. We achieved this correction by averaging the resting grip apertures (after removing outliers) recorded per participant per hand, and subtracting that constant from all associated MGA values. This correction factor allows us to control for slight variations in IRED placement between the hands as well as variations in hand size within participants. In the interest of being complete, ANOVAs were also run on uncorrected data; the significant effects observed and reported below did not change.

### Data Processing

Data were collected via NDI First Principles, all kinematic calculations were performed on unfiltered data using Microsoft Excel 2010, and statistical analyses were completed using PASW Statistics 18.0.0. We determined kinematic parameters using finite differences in the two-step method. Using this method, average speed at time *n* is calculated by determining displacement between times *n−1* and *n+1*, and dividing that displacement by the elapsed time between those two points. The method can be expressed by the formula *v* = [*P*(*n*+1)*−P*(*n−*1) ]/Δt, where *v* is velocity, *P* is position, *n* is a single time point in the output data, and Δt is time elapsed between points *n−*1 and *n*+1. Two participants were missing critical data on greater than 10% of trials due to camera line-of-sight failure, and as such were removed from analyses. The 11 remaining participants were missing critical data on an average of 2.9% (range: 2–7%) of trials. The offending trials were removed from further analysis. Trials were averaged by condition, with 3-way within-subject repeated measures analyses of variance [Hand (LEFT/RIGHT)×Task (EAT/PLACE)×Size (SMALL/MEDIUM/LARGE)] run on condition means. Alpha significance for initial ANOVA results was set at p<.05. Post-hoc comparisons were conducted via paired sample t-tests, with Bonferroni corrections applied where necessary. Estimate of effect size is reported using partial η^2^.

## Results

Significant main effects and interactions are reported below. Between subject means and standard errors of all measurements are reported in Table 1. Significant results are grouped by independent variable.

**Table 1:** Means and standard errors of reach and grasp kinematics.

			MT (ms)	PV (m/s)	DP (%MT)	MGA (mm)	vMGA (mm of SD)
Left	Place	Small	854±29	.659±.040	67.4±1.1	21.66±1.34	2.95±.43
		Medium	839±30	.656±.035	66.8±0.9	24.79±1.49	3.21±.31
		Large	827±31	.679±.041	67.1±1.1	30.71±1.48	4.33±.57
	Eat	Small	858±13	.681±.039	67.1±1.2	21.07±1.59	3.20±.38
		Medium	869±20	.665±.039	68.1±0.9	24.10±1.60	3.63±.23
		Large	838±16	.681±.038	66.6±1.0	29.51±1.47	3.94±.46
Right	Place	Small	865±38	.678±.026	69.0±0.8	19.40±1.18	2.98±.33
		Medium	850±38	.661±.025	68.0±0.8	23.02±1.38	3.50±.33
		Large	870±51	.673±.028	67.6±1.0	28.86±2.03	3.38±.43
	Eat	Small	876±31	.681±.029	68.8±0.5	16.48±1.08	2.64±.35
		Medium	852±38	.674±.030	67.4±1.0	19.57±1.19	2.74±.36
		Large	827±32	.680±.029	67.1±0.6	25.71±1.62	3.18±.37

Units are recorded in column headers.

### Hand

A main effect of hand was observed for MGA [F(1, 10) = 7.902, p = .018, η^2^ = .441], with the RIGHT hand producing significantly smaller MGAs (22.17±1.29 mm) than the LEFT hand (25.31±1.38 mm). No other variables displayed a significant main effect of hand.

### Task

A main effect of task was observed for MGA [F(1, 10) = 19.317, p = .001, η^2^ = .659], with smaller MGAs associated with the EAT task (22.74±1.25 mm) than the PLACE task (24.74±1.21 mm). No other variables displayed a significant main effect of task.

### Size

Main effects of size were observed for MT [F(2, 20) = 7.004, p = .005, η^2^ = .412], PV [F(2, 20) = 6.713, p = .006, η^2^ = .402], DP [F(2, 20) = 6.082, p = .009, η^2^ = .378], MGA [F(2, 20) = 71.485, p<.001, η^2^ = .877], and vMGA [F(2, 20) = 6.042, p = .009, η^2^ = .377]. Larger targets were associated with shorter movement times (with shorter associated deceleration phases), and larger, more variable maximum grip apertures. Grasps directed towards the MEDIUM target achieved significantly lower peak velocities than did those compared to the LARGE target but not the SMALL target. The results of post-hoc analyses are reported in Table 2.

**Table 2:** Means and standard errors of reach and grasp kinematics, collapsed across hand and task.

	MT(ms)	PV(m/s)	DP(%MT)	MGA(mm)	vMGA(mm of SD)
SMALL	863±23‡	.675±.032	68.1±0.8‡	19.65±1.17† ‡	2.94±.19‡
MEDIUM	852±27	.664±.031‡	67.5±0.8	22.87±1.22‡	3.27±.17
LARGE	840±27	.678±.032	67.1±0.8	28.70±1.46	3.71±.19

Main effects of size were discovered for all variables.

† designates a significant difference between the value and value for MEDIUM sized items;

‡ designates a significant difference between the value and value for LARGE sized items.

Significant alphas have been Bonferroni-adjusted for 3 tests (*p*<.0167).

### Hand × Task

**Figure 2:**
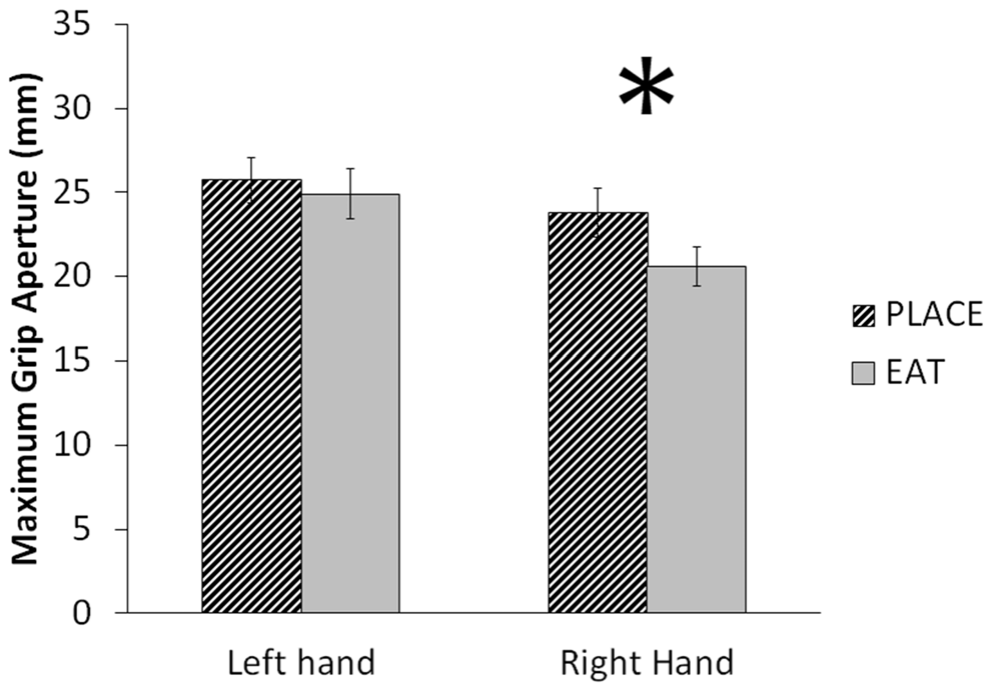
Hand × Task interaction on MGA. Values shown are means+standard errors. PLACE and EAT conditions were significantly different from each other in right-handed movements only; left-handed movements were not significantly affected by task.

**Figure 3:**
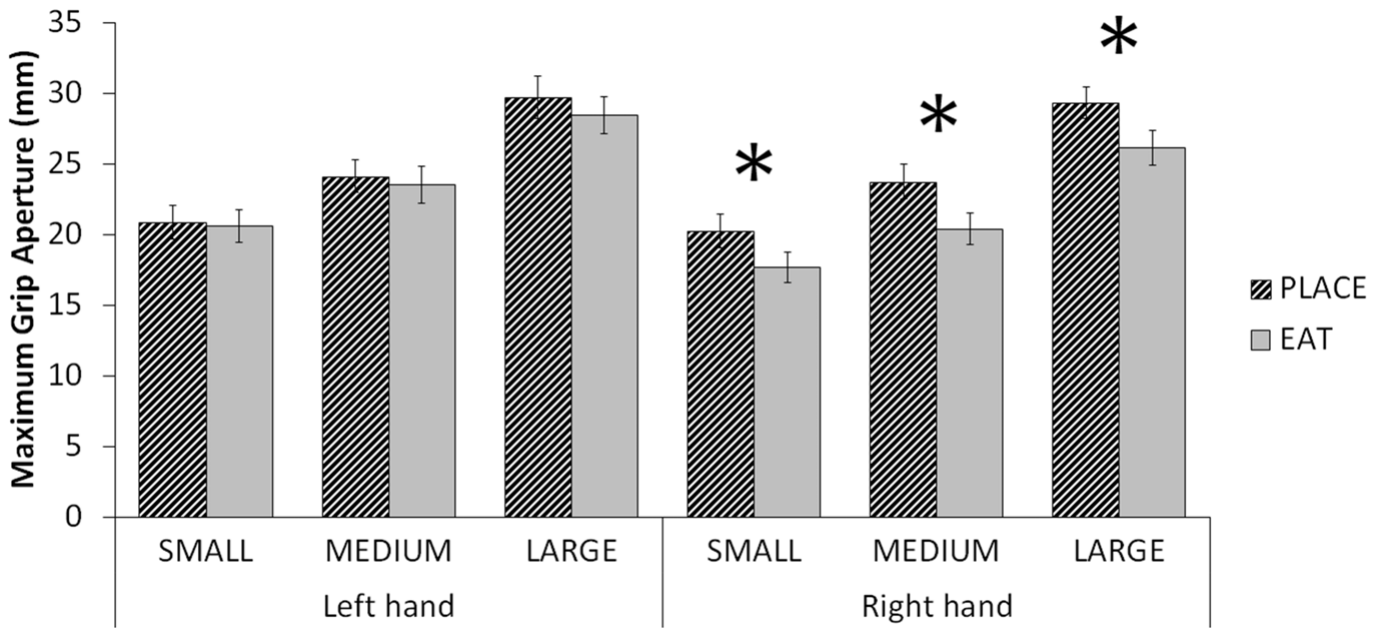
MGA displayed by Hand × Task × Size. The observed Hand × Task interaction (Fig. 2) is consistent across all size conditions. Significance shown (*) has been Bonferroni-adjusted for 6 tests (*p*<.00833).

A significant Hand × Task interaction was observed on MGA, F(1, 10) = 6.887, p = .025, η2 = .408 (Fig. 2). Follow-up paired-samples t-tests revealed that right-handed MGAs in the EAT condition (20.59±1.18) were significantly smaller than those in the PLACE condition (23.76±1.45), t(10) = 5.134, p<.001. Left-handed EAT (24.89±1.48) and PLACE (25.72±1.34) conditions were not significantly different from each other, t(10) = 1.272, p = .232. In fact, MGAs of the right hand for the PLACE condition did not differ from MGAs of the left hand in either condition (p>.15). That is, only right hand MGA for the EAT condition was significantly different from all other conditions (p≤.001). Post-hoc paired-sample t-tests showed that this effect was consistent across all size conditions (Fig. 3). No other Hand × Task interactions were observed.

### Hand × Task × Size

No significant Hand × Task × Size interactions were observed.

### MGA Scaling Analysis

**Figure 4:**
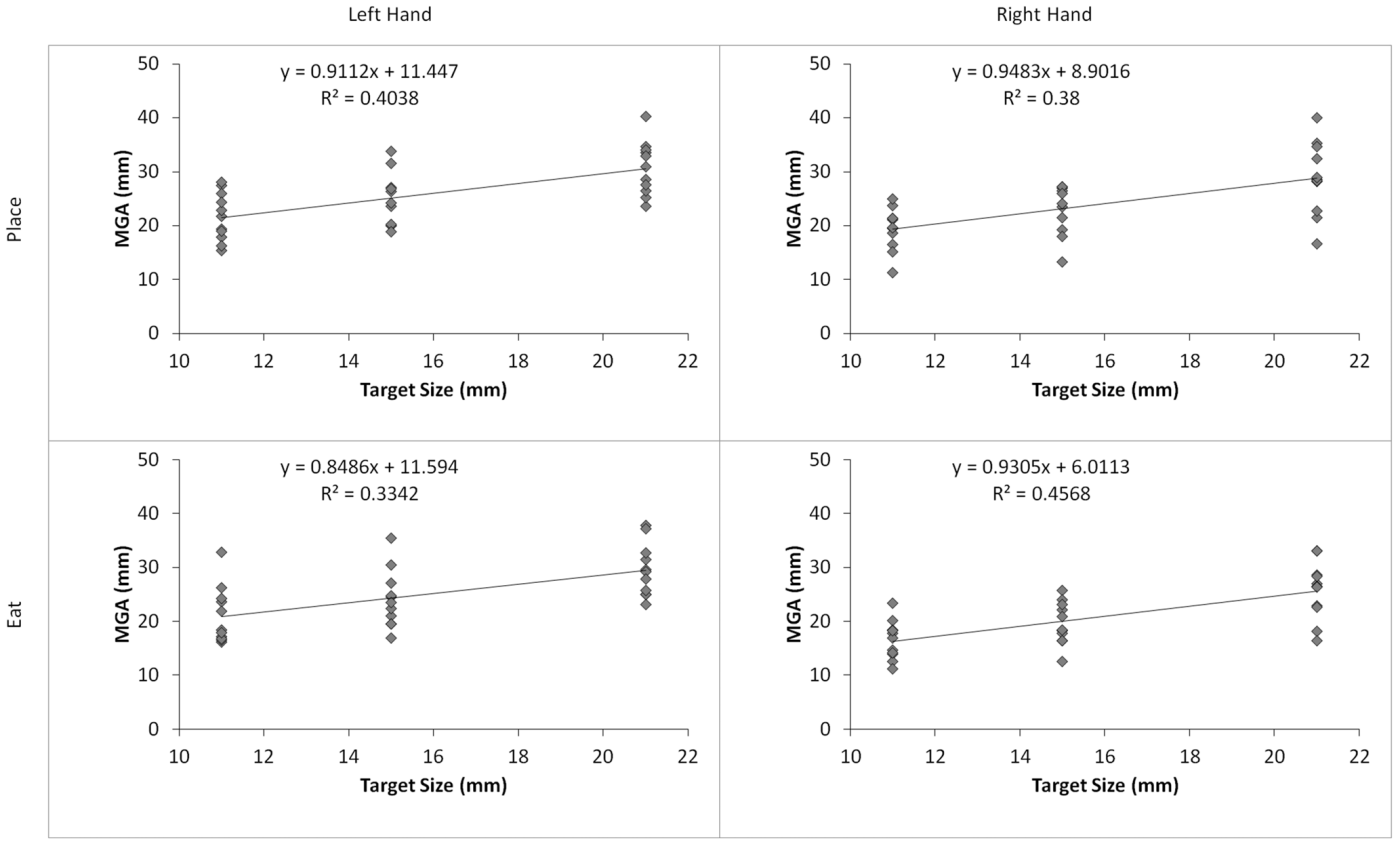
MGA and slope for all Hand × Task conditions. Slopes between conditions were not significantly different.

To further investigate the MGA results reported above, we analyzed the sensitivity of the MGA to changes in target size by plotting each subject’s MGA versus average target size in each hand and task condition (Fig. 4) [32]. To test for differences in scaling, we subjected the regression slopes to a 2 (Hand)×2 (Task) ANOVA [32], [33]. The results of our ANOVA indicate that the slopes were not significantly different between hands (F(1, 10) = 0.307, p = .592, η2 = .03) or tasks (F(1, 10) = 0.777, p = .399, η2 = .072), nor was there a significant interaction (F(1, 10) = 0.15, p = .707, η2 = .015). This finding suggests that the Hand × Task interaction effect observed is not a result of a difference in scaling ability; rather, right hand pre-shaping is simply less wide when the end-goal of the movement is to eat.

## Discussion

While many studies have documented the kinematics of reach-to-grasp actions, few have compared these between hands, and (to the best of our knowledge) none of these have used a grasp-to-eat task. In the current study, participants reached-to-grasp food items in order to either a) eat the item (grasp-to-eat) or b) place the item in a bib located just beneath their chin (grasp-to-place). The bib was chosen for the grasp-to-place task in order to maintain movement trajectory in both the initial approach and the majority of the post-grasp transport phase. Participants completed these tasks with both their left and right hands. Our main finding was that participants prepared a smaller maximum grip aperture when grasping with intent to eat, but only in right-handed movements. This effect was consistent across *all* three sizes of food items, and present in *all* participants. We offer three possible, non-competing interpretations of this finding. First, our results provide behavioural support for the evidence in humans and non-human primates that prehensile movements can differ in their neural correlates based on action intent. Specifically, we differentiate between grasp-to-eat and grasp-to-place actions. Second, the results may be interpreted as evidence that visually-guided grasping can be influenced by experience. And finally, because the grasp-to-eat movement almost certainly predates other types of manipulative actions, we speculate that the current findings may provide evidence for an evolutionary scenario in which handedness stems, at least in part, from a right hand advantage in the grasp-to-eat movement.

The results from the present study differentiated between grasp-to-eat and grasp-to-place actions at the kinematic level. Previous studies have shown that affordances and intentions have significant effects on the kinematics of a movement. Such studies have reported differences in the reach-to-grasp kinematics of throwing and placing actions [13], grasp-to-lift and grasp-to-show actions [34], and grasp-to-feed and grasp-to-manipulate actions [23]. In one previous study participants were asked to bring a cube of sugar to the mouth or to a mouth-like aperture [23]. Consistent with that study, we found an effect of task on grasp kinematics wherein MGAs were smaller when the object was to be placed in the mouth, rather than simply in a location near the mouth. Unlike Ferri et al., who reported differences in movement time and deceleration time duration, however, we did not observe an effect of task in any other measures. This suggests that the smaller MGA for the grasp-to-eat movement in the current study is not due to differences in other kinematic parameters. Although numerous studies have explored the effects of intentions on movement kinematics, few have yet investigated whether these effects are conserved in left-handed movements (cf. Armbruster & Spijkers, 2006). Ours is the first study to demonstrate that action intent affects grasping movements differently according to the hand used. Specifically, we show that the right-handed grasp-to-eat movement is more accurate (i.e., produces a smaller margin of error within hand pre-shaping) than both the right-handed grasp-to-place movement, and indeed left-handed movements of both types. It should be noted that this advantage was not due to more precise *scaling* of right-handed grasping (MGA slopes between the EAT and PLACE conditions revealed similar grip aperture scaling for both hands), but rather the right-hand grasps were produced with a smaller margin of error when the target was to be eaten. This could be considered as an advantage for two reasons. First, numerous reach-to-grasp studies have shown that MGA is remarkably sensitive to target uncertainty such that increases in uncertainty are linked with larger MGAs. For example, if vision of the target or reaching limb is removed at the beginning of a grasp (introducing target and movement uncertainty), MGA is larger than in movements directed to the same target with full visual feedback [26], [35]–[40]. This increase in MGA is even larger when a brief delay is introduced between vision restriction and movement onset such that the grasp is initiated and completed entirely from memory [26], [41], [42]. Thus, target uncertainty can result in the production of larger MGAs, presumably as a means of compensation via wider margins of error [36]. Second, smaller MGAs could be considered more efficient because peak grip-closing velocity, grip-closing time and metabolic energy requirements are reduced when the MGA more closely approximates the absolute size of the target [43].

Kinematic differences between grasp-to-eat and grasp-to-place actions may stem from differences in their neural correlates. Electrophysiological studies on non-human primates have shown that different cortical regions are responsible for grasp-to-eat versus grasp-to-manipulate actions. In an influential account of motor cortex organization and function, Graziano [44] described several experiments which demonstrate a motor cortex organized not around specific control of muscles, but rather around producing complex coordinated behaviours. When a macaque’s motor cortex was directly stimulated using long electrical pulses of 500–1000 ms (the approximate duration of a typical prehensile movement), the macaque produced behaviourally-relevant actions. Long-train stimulation yielded reaching movements [45], grasp-to-manipulate movements [46], and hand-to-mouth grasping movements [45]. These movements were context-relevant and goal-oriented. Notably, the grasp-to-manipulate movement and the hand-to-mouth grasping movement were produced by stimulating two distinct anatomical locations [44]. Single-neuron recording studies performed by Fogassi et al. [47] and Bonini et al. [48], [49] have identified task-specific neurons in both the inferior parietal area PFG and ventral premotor area F5 in macaques. The researchers have shown that individual neurons respond differentially to the *purpose* of the grasp (*place* vs. *eat*) rather than to the object being grasped [47], the pre- or post-contact kinematics of the action [47], [48], or the hand shape required for successful prehension [49]. These results, along with those of others [50], [51], suggest that the grasp-to-manipulate and grasp-to-eat actions are supported by neural networks with discrete origins. Although unknown, it is probable that similar distinctions exist in the human brain. While the invasiveness of these studies make them infeasible to perform with human participants, and the limitations of fMRI make reach-to-eat tasks difficult, researchers have nevertheless been able to highlight circuits in the human parietal cortex that respond selectively based on action intent [52]–[54]. For example, using fMRI, researchers have identified discrete regions activated by movements that share similar kinematics but differ in their purpose. Selective activation of the superior parieto-occipital cortex (SPOC) during planning and execution of aiming movements [55], and anterior intraparietal sulcus (aIPS) activation during grasping [56]–[58] have been shown. Most recently, Gallivan et al. [58] found that when participants were given a choice to either grasp or touch one of several target cubes, both the chosen target and movement intention could be accurately predicted from activation of specific locations within the aIPS. These studies show that although movements may share similar kinematics, it is the actor’s intent that will determine the neural origin of the movement.

In terms of experience, it is possible that the right-hand advantage found in this study relates to the increased amount of practice executing the grasp-to-eat movement with this hand. As mentioned in the introduction, the right-hand preference for grasp-to-eat movements develops several years earlier than does the preference for grasp-to-place movements. In fact, the hand-to-mouth/grasp-to-eat movement is one of the first movements to arise in human infants [59]. Fetuses have been observed to make this movement in the womb for the purpose of thumb-sucking and furthermore have demonstrated a right-hand preference for this and other hand-to-face movements [60]. Behaviourally, a right-hand preference for the grasp-to-eat movement has been demonstrated in young infants [61] and has been contrasted with the grasp-to-place movement [10]. These studies have shown that children as young as one year of age prefer to use their right hands for grasping, but *only* when performing the grasp-to-eat movement. When children are required to grasp other, non-edible objects (e.g. toys or blocks), a right hand preference is not visible until children reach 4 years of age [8]–[10]. These findings demonstrate that right hand preference for the grasp-to-eat action develops considerably earlier than the right hand preference for other manipulative movements. These additional years of experience, which coincide with a critical period in the development of coordination and consistency in the reach-to-grasp movement [62]–[64], might be responsible for the right hand kinematic advantage in the grasp-to-eat action. Furthermore, practice has also been shown to alter the kinematics of both reach [65] and reach-to-grasp movements [66] in adulthood. For example, one study demonstrated that awkward grasps (grasps made using the thumb and ring finger) are initially sensitive to a visual illusion. However after one hour of practice with the awkward grasp for three consecutive days, the effect of the illusion on the awkward grasp was reduced to an extent where the awkward grasp pre-shaping resembled that of the more common pincer grasp [66]. Importantly, this reduction was only observed for right-handed movements; left-handed grasp scaling remained susceptible to the effects of the illusion regardless of practice. The authors speculated that the right hand control system is able to incorporate previous experience into hand pre-shaping for grasping purposes. This ability may be the source of smaller right-hand MGAs observed in the current study. In sum, if we have more experience with our right-hand for the grasp-to-eat movement during development, and this practice results in both increased coordination and increased target certainty, then it is reasonable to speculate that we would produce grip apertures with smaller margins of error in the grasp-to-eat movement.

While the current study investigated this effect exclusively in right-handed participants, we predict that left-handed individuals would also demonstrate smaller MGAs in the grasp-to-eat movement. However, as left-handers represent a more heterogeneous group with respect to hemispheric lateralization [67], [68], we would expect that the MGA effect described here would not be consistently confined to the dominant hand between participants. As has been demonstrated before, while some left-handers prefer the use of their dominant left hands for grasping, a subset of left-handers exhibit a preference for their non-dominant right hands for grasping [27], [69]–[71]. It is tempting to speculate that this subset would resemble right-handers in their grasp-to-eat behaviour, producing smaller MGAs when using their right hands for this task. Meanwhile, left-handers who prefer to grasp with their dominant hands would express the reverse behaviour; that is, they would produce smaller grasp-to-eat MGAs when using their left-hands. Future studies will investigate this possibility.

Finally, when considered from an evolutionary perspective, the results of this study may provide insight into why 90% of the global population is right-handed. It has long been speculated that animal prehension evolved from grasp-to-eat actions [28], [29], [72], [73]. While handedness in humans is often measured by our lateralization of tool-use, some researchers argue that the development of skilled praxis in primates stems from early behaviour in food preparation or capture [28]. One theory, known as the postural origins theory [72], [74], posits that the evolution of right-handedness in humans and other great apes began from a right-hand specialization for postural maintenance, co-opted for foraging when prosimians evolved to utilize a more upright stance. The right-hand/left-hemisphere system, then specialized for the production of the precise grip-forces necessary to maintain arboreal positions, was perfectly suited for the fine manipulations necessary to husk nuts, peel fruit, and grasp other fragile food when bimanual foraging became possible [72]. This theory is consistent with observations of population level right-hand preference for grasp-to-eat actions in several species, including chimpanzees (*Pan troglodytes*) [75]–[77], gorillas (*Gorilla gorilla*) [78], bonobos (*Pan paniscus*) [78], and humans (*Homo sapiens*) [10], [75]. Additional support for this theory comes from the similarities between human and chimpanzee grasping behaviour. In humans, lateralization of prehension is predominantly rightward-biased, especially for precision grips [69], [70], [75] and grasp-to-eat actions [10], [75]; chimpanzees show a similar pattern of right-hand preference for grasp-to-eat actions, with right-hand use increasing with the precision requirements of the grasp [75]. Furthermore, the development of the precision grip from juvenile to adulthood follows a similar trajectory in both humans and chimpanzees. When grasping small objects, members of both species tend to use the less precise whole-hand or power-grips as juveniles, gradually shifting to more controlled precision grips as adults [75]. This observation further strengthens the position that handedness is driven by the right hand’s ability for precision grasping. All told, these studies suggest that left-hemisphere specialisation for precise visually-guided movements in humans has shared origins with the right hand preference for grasp-to-eat movements observed in chimpanzees and other great apes.

As numerous studies have explored the possible link between the lateralization of praxis and language [79], [80] (namely, that the left-hemisphere lateralization of gesture developed as a predecessor to language in *Homo sapiens*), it is tempting to speculate that the hand preference for the grasp-to-eat action is a good candidate for the neural basis upon which hand preference for praxis (i.e. tool use and gesturing) and eventually language evolved (for review, see Corballis [79]). Recent work by Pulvermüller and colleagues [81], [82] has demonstrated that language and motor regions are cortico-cortically linked; hearing or reading words coupled with specific body parts (“kick” and “lick,” for example) differentially activates the motor cortex areas associated with those parts of the body [82], and that this ‘semantic somatotopy’ is critically related to higher level language comprehension [81]. Regarding asymmetries, other investigations have shown that the degree of left-hemisphere lateralization for language is linearly related to the degree of right-hand preference for everyday activities [83], particularly that for precision grasping [70]. As has been argued before [66], [70], the lateralization of precision grasping may in fact predate the development of specific circuits for praxis and language, evidenced by the aforementioned left-hemisphere specialization for precision grip observed in chimpanzees [76], [77].

### Conclusion

In conclusion, the results of the current investigation show a kinematic dissociation between the grasp-to-eat and grasp-to-place actions. This dissociation, however, was only present within right-handed movements. More importantly, the results demonstrate a right-hand advantage for the grasp-to-eat action. We speculate that this advantage could have served as the basis for the well-known right hand dominance for manual functions. An examination of the neuroanatomical and neurophysiological correlates of this finding may provide fresh insight into the evolutionary origins of handedness in humans.
